# Human *BLCAP* transcript: new editing events in normal and cancerous tissues

**DOI:** 10.1002/ijc.25022

**Published:** 2009-11-11

**Authors:** Federica Galeano, Anne Leroy, Claudia Rossetti, Irina Gromova, Philippe Gautier, Liam P Keegan, Luca Massimi, Concezio Di Rocco, Mary A O'Connell, Angela Gallo

**Affiliations:** 1 Ospedale Pediatrico Bambino Gesù, Piazza S. Onofrio4, 00165 Rome, Italy; 2 MRC Human Genetics Unit, Institute of Genetics and Molecular Medicine, Western General HospitalEdinburgh EH4 2XU, United Kingdom; 3 Institute of Cancer Biology, Danish Cancer Society, Danish Centre for Translational Breast Cancer ResearchCopenhagen, Denmark; 4 Pediatric Neurosurgery Policlinico Gemelli, Largo Agostino Gemelli8, 00168 Rome, Italy

**Keywords:** RNA editing, ADARs, BLCAP, cancer, dsRNA structure

## Abstract

Bladder cancer-associated protein (BLCAP) is a highly conserved protein among species, and it is considered a novel candidate tumor suppressor gene originally identified from human bladder carcinoma. However, little is known about the regulation or the function of this protein. Here, we show that the human *BLCAP* transcript undergoes multiple A-to-I editing events. Some of the new editing events alter the highly conserved amino terminus of the protein creating alternative protein isoforms by changing the genetically coded amino acids. We found that both ADAR1 and ADAR2-editing enzymes cooperate to edit this transcript and that different tissues displayed distinctive ratios of edited and unedited *BLCAP* transcripts. Moreover, we observed a general decrease in *BLCAP*-editing level in astrocytomas, bladder cancer and colorectal cancer when compared with the related normal tissues. The newly identified editing events, found to be downregulated in cancers, could be useful for future studies as a diagnostic tool to distinguish malignancies or epigenetic changes in different tumors.

In eukaryotes, mRNA transcripts are extensively processed by post-transcriptional events such as alternative splicing and RNA editing to generate different mRNAs from the same gene. In mammals, the most common type of editing involves the conversion of adenosines into inosines.^1^,^2^ A-to-I RNA editing has the potential to change the RNA sequence, in fact, inosine is read as guanosine by both the translation machinery^3^ and the splicing machinery.^4^ Furthermore, the inosine does not pair with uracil but instead with cytosine, changing the (A–U) base pair into an (I:U) mismatch.^5^ Therefore, A-to-I editing has also the potential to change the secondary structure of RNA molecules. This type of editing is catalyzed by a family of enzymes called adenosine deaminases acting on RNA (ADARs). ADARs have a conserved domain arrangement that include 2 or 3 dsRNA-binding domains (RBDs) at the amino terminus and a highly conserved deaminase domain at the carboxy terminus.^1^,^6^ Three ADARs have been identified. ADAR1 and ADAR2 are widely expressed and active enzymes however, ADAR3 is present exclusively in the brain and is inactive *in vitro* assays.^7^,^8^

The total amount of inosines present in mammalian tissues was found to occur at a higher frequency than previously estimated.^9^ Furthermore, it has been shown that the majority of the A-to-I substitutions occur not within the coding region of mRNAs but in noncoding RNA sequences particularly within the untranslated regions (UTRs).^10^–^12^ Transcripts edited within their coding sequence are mainly expressed in the central nervous system (CNS) and, as a consequence, alterations in RNA editing are often implicated in pathologies that affect the CNS such as epilepsy,^13^ depression,^14^ ALS^15^ and brain tumors.^16^–^20^

RNA-editing levels change during neuronal-cell differentiation and vary within different regions of the brain.^18^,^21^–^23^ However, little is known about how and if ADARs are differently modulated *in vivo* in tissues other than the brain. This could be important as these enzymes are widely expressed and their activity is essential in tissues of non-neurological origin.^24^ Therefore, identification of additional ADAR substrates in these tissues would assist in understanding the regulation of the ADAR enzymes. Recently, considerable effort has been spent in this area of research.^10^,^25^–^27^ We focused our attention on a transcript that is ubiquitously expressed that encodes bladder cancer-associated protein (BLCAP) also known as BC10 protein (bladder cancer-10 kDa protein) that is an 87 amino acid protein with 2 hypothetical transmembrane regions (TM).^28^,^29^ Importantly, it has been shown that the mRNA of *BLCAP* is downregulated in bladder invasive carcinoma^28^ and in renal cell carcinoma^30^ and in primary cervical carcinoma,^31^ underlining the potential onco-suppressive role of this protein. Nevertheless, the biological role of BLCAP has not yet been elucidated.

Here, we identify novel editing sites in the human *BLCAP* transcript in both coding and noncoding sequences. We found that the new editing events were present in the different human tissues studied and that the percentage of editing varies strongly depending on the tissues analyzed. We found that both ADAR1 and ADAR2 play a cooperative role in editing this substrate. Our work opens up new possibilities in studying the regulation of ADARs and their activity in different cell types and tissues by monitoring the editing in the *BLCAP* transcript. Moreover, we report that editing of the *BLCAP* transcript is downregulated in cancerous tissues that could be of importance for future-improved diagnosis and prognosis in a variety of different cancer types.

## Material and Methods

### Tissues and cell lines

Tissue and cell-line samples used in this study were identified by progressive identification numbers, ID 1–21:

(ID1) human heart from Clonthech, BD Biosciences; (ID2) human bladder from Clonthech, BD Biosciences; (ID3) normal blood lymphocytes, patient 2630; (ID4) normal epithelial tissue from colonic normal mucosa, patient 2630; (ID5) skin fibroblast from patient 2630; (ID6) white matter, patient n.22; (ID7) human bladder carcinoma grade II, (ID8) human bladder carcinoma grade III; (ID9) human bladder carcinoma grade III 727-1; (ID10) tumorigenic human bladder cell line (TCC) HU 609 (kind gift from Dr. Toben Ørntoft, Denmark); (ID11) human bladder cell line (TCC) HCV 29 (kind gift from Dr. Toben Ørntoft, Denmark); (ID12) human bladder epithelial invasive carcinoma cell line (TCC) T24 (ATCC); (ID13) colorectal cancerous epithelial tissue from a polyp developed by patient 2630; (ID14) colorectal cancerous epithelial tissue from patient 425; (ID15) epithelial colorectal cancer cell line from patient 425 (MYH deficient), VACO425; (ID16) epithelial colorectal cancer cell line CACO2; (ID17) epithelial colorectal cancer cell line HRT18; (ID18) pediatric astrocytoma grade IV patient n.22; (ID19) astrocytoma cell line U118 MG (ATCC); (ID20) astrocytoma cell line A172 (ATCC); (ID21) HeLa cell line (ATCC). All the samples identified by ID numbers were tested for RNA-editing levels on a *BLCAP* transcript after RT-PCR, and individual cDNA clones were sequenced for each sample. Fresh tumor and control tissues were dissected and placed on dry ice until required. Tumor cell lines were maintained in Dulbecco's modified Eagle's medium (DMEM) supplemented with 10% fetal calf serum (FCS) and 1% penicillin–streptomycin.

### Extraction of RNA

Total RNA was isolated with TRIzol Reagent (GIBCO) from tumors, control tissues and cultured cell lines according to the manufacturer's instructions.

### RT-PCR from the different tissues and cell lines

One microgram of purified total RNAs pretreated with DNase I was incubated at 70°C in the presence of 1 μM of the appropriate specific reverse primer. The hBLCAP-3′RT primer 5′-CCGTCTTCTGCTTCCTTGGAAAGCTAACAG-3′ was used to reverse transcribe part of the 3′UTR, the coding sequence and the 5′UTR of *BLCAP*. The 3′RT primer 5′-GAAGAGAAAAGTCAACACAACAGACAAACA-3′ was used to reverse transcribe the pre-mRNA that includes the intron. After a 30-min incubation the samples were placed on ice for 5 min. Reactions were then performed in the presence or absence of M-MLV reverse transcriptase, RNase H Minus (Promega), following the suppliers instructions and incubated for 2 h at 42°C. The cDNA obtained was amplified by PCR with the expand high fidelity PCR System (Roche). hBLCAP-5′F 5′-GATCCCTGCTGCCTTGGTGATCCCGGG CTG-3′ and hBLCAP-3′RT were used to amplify a fragment of 479 bp encompassing the 5′UTR, ORF and 39 bp of the 3′UTR. hBLCAP-intron5′ 5′-CTTAAAATATTTCGAGGCTG TAGCTGCCTC-3′ and hBLCAP-intron 3RT were used to amplify a fragment of 580 bp within the intron that was predicted by bioinformatics to base pair with exon 2 and thus form a double-stranded substrate for the ADAR enzymes. Thirty cycles of the following PCR program were used: denaturing 30 s at 94°C, annealing 30 s at 58°C and extension for 1 min at 68°C. Specific products were gel purified (Qiaquick, Qiagen) and directly sequenced or cloned into pGEM T-easy vector (Promega) and transformed into *Escherichia coli* and sequenced with T7 and Sp6.

### Analysis of RNA editing

Sequences were analyzed with Seqman software (Lasergene), and direct sequencing was performed on cDNA pools. Editing was calculated as previously described^32^ or the PCR products were subcloned into the T-easy vector (Promega), and ∼30–50 individual cDNA clones were sequenced for each sample. A–G changes in the individual clones were analyzed. For each sample, 2–3 independent RT-PCR reactions were performed.

### Astrocytoma and HEK 293T cell lines

cDNA, encoding the shorter and more active human isoform ADAR2a and the human ADAR1 long isoform (ADAR1 150kDa), were subcloned into pEGFP C3 vector (V-EGFP) (Clontech) with in-frame enhanced green fluorescent protein (EGFP) at the amino terminus. The HEK 293T cell line was cultured in DMEM.

HEK 293T cell line was chosen for the assessment of EGFP-ADAR2 and EGFP-ADAR1-editing activity on the *BLCAP* transcript, as the endogenous editing activity in this cell line is low or undetectable.^18^,^20^,^33^ The HEK 293T cell line was cultured in DMEM and ∼7 × 10^5^ HEK 293T cells at 60% confluence were transiently co-transfected using lipofectamine 2000 (Invitrogene), with either 4 μg of EGFP-ADAR2 or EGFP-ADAR1 in the constant presence of 3 μg of GluR-B minigeneB13 (miniB13), which was used as an exogenous editable substrate. The miniB13 encodes a portion of the editing-competent murine glutamate receptor GluR-B gene that includes the Q/R and +4 site that are edited mainly by ADAR2 and the hot-spot 1 site mainly edited by ADAR1.^34^,^35^ Forty-eight hours after transfection, total RNA was extracted from the cells using TRIzol Reagent and the transfection efficiency tested by real-time PCR experiments. We then extracted the total RNA and performed RT-PCR with specific oligonucleotides for both the endogenous *BLCAP* mRNA with the oligonucleotides hBLCAP-5′F and hBLCAP-3′RT and the exogenous miniB13 with specific miniB13 oligonucleotides, mini13 FW 5′-TTTAGCCTATGAGATCTG GATGTGC-3′ and miniB13 Rev 5′-GAAGATTCGTA GAATTTAAACG-3′. The endogenous *BLCAP* and the exogenous miniB13 were amplified, and the editing level calculated by direct sequencing of the cDNA pools as previously described^32^ (as for the miniB13) or the PCR products were subcloned into the T-easy vector (Promega) (as for the *BLCAP*). After subcloning ∼30 individual cDNAs were sequenced and A-G changes in the individual clones were analyzed. For each sample, 2 independent RT-PCR reactions were performed. Human astrocytoma cell lines A172 (ATCC CRL-1620™) and U118 (ATCC HTB-15™) were grown in DMEM supplemented with 10% FCS (Invitrogen) plus antibiotics. Astrocytoma cell lines were transfected with pEGFP and EGFP-ADAR2 (8 μg) using lipofectamine 2000 (Invitrogene). After 48 h, the cells were collected and the endogenous *BLCAP* cDNA analyzed for RNA editing.

### RNA folding

Mfold web server was used for nucleic acid folding and hybridization prediction.^36^,^37^

## Results

### Human *BLCAP* transcript is edited in its coding and noncoding sequences

**Figure 1:**
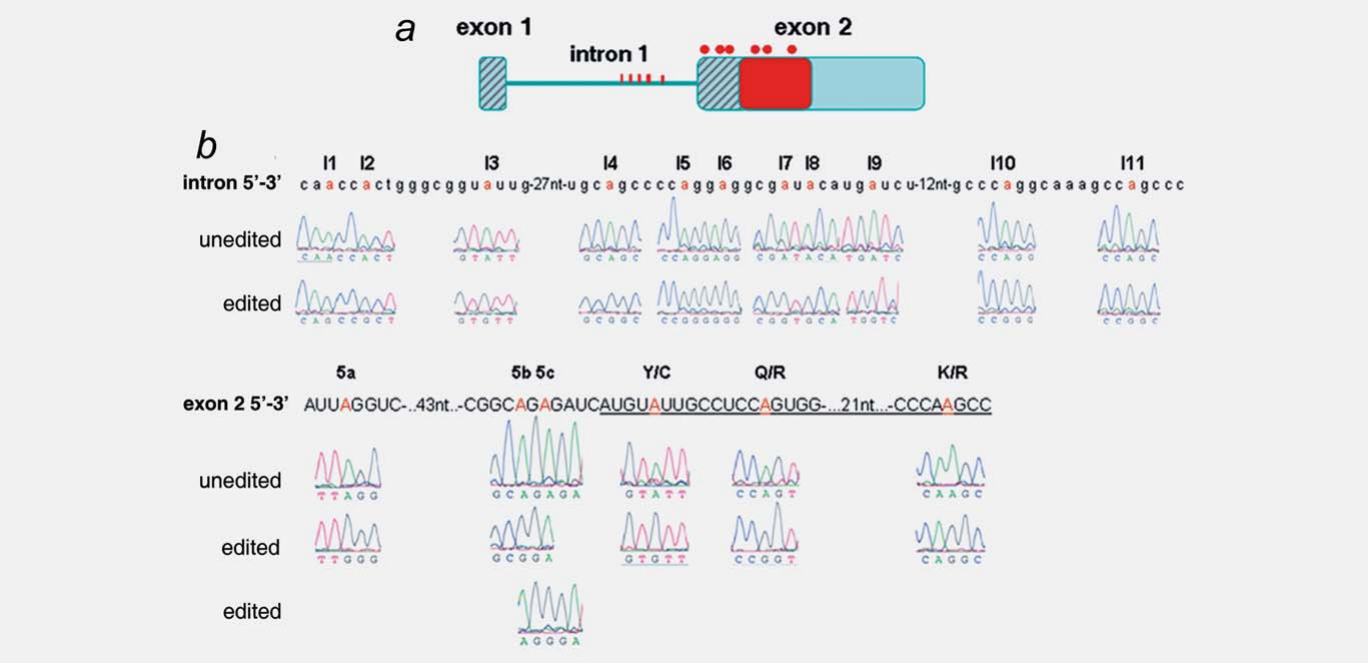
New editing events in the human *BLCAP* transcript. (*a*) Schematic representation of the *BLCAP* pre-mRNA. Exon 1 encoding the 5′UTR is represented in light blue with gray bars followed by the intron that divides the 5′UTR in 2. Exon 2 encodes the remaining 5′UTR (light blue with gray bars), the coding sequence (red rectangle) and the 3′UTR (light blue). Red dots indicate the editing sites in *BLCAP* mRNA (5′UTR and coding sequences). Red lines indicate the editing sites identified within the intron. (*b*) Sequences of *BLCAP* pre-mRNA. Adenosines in red indicate the edited sites in the intron (small letters) and exon 2 (capital letters). The underlined sequence corresponds to the coding region. The intron was found to be edited at 11 positions referred to as I1 to I11 from 5′ to 3′. The 5′UTR was edited at 3 positions referred to as: 5a, 5b and 5c. In the coding sequence, the edited positions are named according to the amino acid change they produce: Y/C, Q/R and K/R. Underneath, the sequences are shown the chromatograms of single clones after sequencing analysis of human *BLCAP* pre-mRNA in normal bladder tissue. As these sequences are cDNAs, the inosines are read as guanosines and the uridines as thymidines. The percentage of the editing events identified in the bladder tissue at different sites is shown in Table 3.

**Figure 2:**
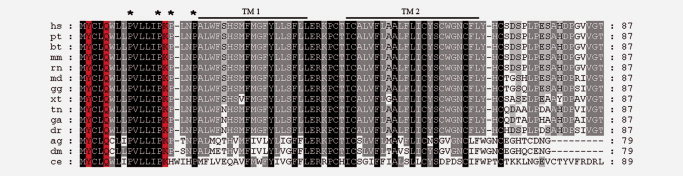
Multiple alignments of BLCAP proteins. Multiple alignments of BC10 proteins. Initials correspond to hs, *Homo sapiens*; pt, *Pan troglodytes*; bt, *Bos taurus*; mm, *Mus musculus*; rn, *Rattus norvegicus*; md, *Monodelphis domestica*; gg, *Gallus gallus*; xt, *Xenopus tropicalis*; tn, *Tetraodon nigroviridis*; ga, *Gasterosteus aculeatus*; dr, *Danio rerio*; ag, *Anopheles gambiae*; dm, *Drosophila melanogaster*; ce, *Caenorhabditis elegans*. Identical amino acid conservation among species is indicated in black. Residues identical in most of the species analyzed are indicated in gray. Indicated in red are the amino acids found to be edited. Black lines above the alignment indicate the hypothetical transmembrane domains (TM). Stars indicated the proline-rich motif at the N-terminus.

The human *BLCAP* gene is located on chromosome 20 and comprises 2 exons separated by an intron (Fig. 1*a*). Exon 1 encodes a 5′ sequence of the 5′UTR. Exon 2 includes the remaining sequence of the 5′UTR, the coding region and the 3′UTR. The coding sequence of the *BLCAP* gene is therefore intronless (Fig. 1*a*). Editing has previously been reported to occur in the human *BLCAP* transcript changing the second amino acid of the protein at the Y/C editing site.^38^ We wondered if other editing events were present in the human *BLCAP* transcript that could increase the number of protein isoforms. For this purpose we amplified the *BLCAP* pre-mRNA from human bladder with specific oligonucleotides as *BLCAP* is particularly abundant in this tissue, and we performed sequence analysis on pools as well as on single clones. We found that the *BLCAP* pre-mRNA from the human bladder undergoes multiple editing events in the coding, 5′UTR and intronic sequences. New editing events were found within exon 2 (Fig. 1*a*, red dots) and within intron 1 (Fig. 1*a*, red lines). The human *BLCAP* intron was edited in 11 specific sites that we term I1–11 starting from the 5′ to the 3′ (Fig. 1*b*). The 5′UTR, which lies within exon 2, was also edited at 3 positions; 5a, 5b and 5c (Fig. 1*b*). Most importantly, we found 3 editing events within the coding region that changed the amino acid composition of the protein. The tyrosine in position 2 of the protein was converted into a cysteine (Y/C site) as previously reported.^38^ In addition, we found 2 new editing events that converted the glutamine at position 5 to arginine (Q/R site) and the lysine at position 15 to arginine (K/R site) (Fig. 1*b*). Both the new editing events, identified in the coding sequence, inserted a positively charged amino acid within the BLCAP amino terminus (Fig. 1*b*). The percentage of the editing events identified in the bladder tissue at different sites is shown below. Multiple alignments in different organisms of the BLCAP protein showed that this protein is highly conserved from humans to *Drosophila melanogaster* and *Caenorhabditis elegans*, particularly, at its amino-terminus, suggesting that it may have a conserved biological role (Fig. 2). RNA editing can alter this highly conserved portion of the protein by changing 3 different amino acids that can produce up to 8 different protein isoforms (Fig. 2).

### Both ADAR1 and ADAR2 enzymes can edit *BLCAP* transcript in HEK 293T cells

**Figure 3:**
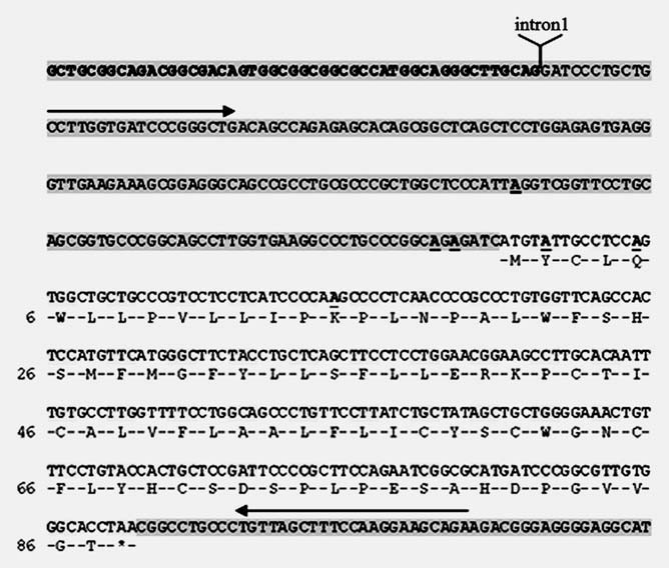
RNA editing in *BLCAP* mRNA. Schematic representation of human *BLCAP* mRNA. Exon 1 is depicted in bold. The remainder is exon 2 that encodes all the editing positions present in the mature *BLCAP* RNA, 3 within the 5′UTR and 3 within the coding sequence. The amino acid sequence is below the nucleotide sequence. The adenosines that are edited are bold and underlined. Arrows identify the position of the oligonucleotides hBLCAP5′F and hBLCAP3′RT used for the amplification of *BLCAP* after RT-PCR.

As *BLCAP* transcript is edited at multiple sites, the question therefore arose as to which ADAR editing enzyme was responsible for these editing events. We focused our attention on the editing sites that are present in the *BLCAP* mRNA as editing in the 5′UTR and coding sequences could affect both protein synthesis and function.^2^,^39^ To address this question we transfected the HEK 293T cell line with either EGFP-ADAR1, EGFP-ADAR2 or the empty vector. The HEK 293T cell line is an ideal system for an editing assay as this cell line displays a low level of an endogenous editing activity.^18^,^33^,^40^,^41^ To ensure comparable transfection efficiency, the percentage of EGFP-positive cells was determined by flow-cytometric analysis (data not shown). We analyzed ADAR1 and ADAR2 expression levels by real-time PCR (Fig. 1S, panel A). To monitor the editing activity of the transfected ADAR enzymes, we also cotransfected the miniB13 that encodes a portion of the editing-competent murine GluR-B gene that carry specific sites for both ADAR1 and ADAR2 (Fig. 1S, panel B). *BLCAP* was amplified with the oligonucleotides hBLCAP5′F and hBLCAP3′RT as shown in Figure 3, and products subcloned into the T-easy vector (Promega) and ∼30 individual cDNA clones were sequenced and results are shown in Table 1.

**Table 1:** Editing of endogenous BLCAP mRNA, within EXON 2 in HEK 293T cells transfected with either ADAR2 or ADAR1

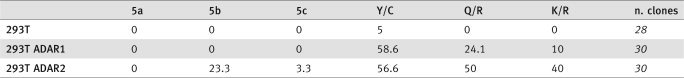

Editing levels are expressed as percentages. In italics is the number of *BLCAP* clones that were sequenced.

We found that none of the 3 editing sites within the 5′UTR (5a–c) were edited by ADAR1, whereas ADAR2 edited the 5b site (23.3 %) as well as the 5c site (3.3%) (Table 1).

The Y/C site displayed a high level of editing (∼60%) (Table 1) in the HEK 293T cell line overexpressing either ADAR1 or ADAR2. Using the same cell assay, we found that ADAR1 also edits the Q/R site to ∼24% but at a very low level the K/R site, whereas the ADAR2 enzyme was able to edit both the Q/R (∼50%) and K/R (∼40%) sites to a comparable efficiency (Table 1). Low/undetectable editing level was found in HEK 293T transfected with the pEGFP empty vector. Therefore, we can conclude from this assay that both ADAR1 and ADAR2 can edit all the sites present in the *BLCAP* mRNA coding sequence, with ADAR2 showing a higher editing efficiency at most sites.

### *hADAR2* edits the *BLCAP* mRNA in astrocytoma cell lines

To further analyze if ADAR2 was responsible for editing the endogenous *BLCAP* transcript within the coding sequence we used a different cell line. Astrocytoma cell lines have a very low-endogenous ADAR2 editing activity as measured by editing of endogenous substrates such as GluR-B and GluR-6 transcripts.^18^ Therefore, we used the astrocytoma cell lines (U118 and A172), both untransfected or transfected with the pEGFP-ADAR2 construct or with pEGFP as a negative control.

When we analyzed the editing of the endogenous *BLCAP* transcript in astrocytoma cell lines, we observed a baseline editing activity of 0–4.2% at the Y/C, Q/R and K/R sites in all 4 control cell lines (U118, U118 EGFP, A172 and A172 EGFP) (Table 2).

**Table 2:** Modulation in *BLCAP* editing in astrocytoma cell lines

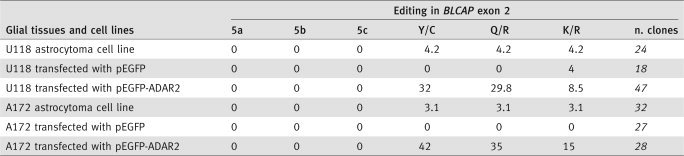

Editing levels are expressed as percentages. In italics is the number of *BLCAP* clones that were sequenced.

**Figure 4:**
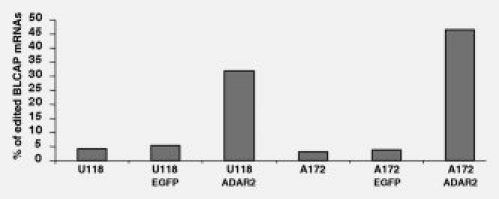
Increased editing activity observed on *BLCAP* mRNA. A172 and U118 astrocytoma cell lines were transfected with EGFP empty vector or a vector expressing EGFP-ADAR2. The editing events present in *BLCAP* mRNA are expressed as a percentage (*y*-axis). On the *x*-axis are the untransfected astrocytoma U118 and A172 cell lines, the cell lines transfected with EGFP vector and with EGFP-ADAR2. ADAR2 increased the number of *BLCAP* transcripts undergoing editing in both cell lines.

As ADAR2 can edit all 3 sites within the coding region of *BLCAP* in HEK 293T cells (Table 1), we expected an increase in editing levels at these sites in astrocytoma cell lines transfected with EGFP-ADAR2. We observed an increase in editing activity at the Y/C site (from 0–4.2% to 32–42%), Q/R site (from 0–4.2% to 30–35%) and K/R site (from 0–4.2% to 8.5–15%) (Table 2). The 3 editing sites within the 5′UTR showed no editing. We found that ∼5% of the total *BLCAP* transcripts were edited in the untransfected or control astrocytoma cell lines transfected with pEGFP. However, when EGFP-ADAR2 was overexpressed, the level of the editing per transcript increased by 6-fold in the U118 cell line and 10-fold in the A172 cell line (Fig. 4). As ADAR2 can edit all 3 sites within the coding sequence of *BLCAP*, this enzyme could strongly decrease the amount of the unedited BLCAP isoform in a cell.

### Co-ordination of intronic and exonic editing events of *BLCAP* pre-mRNA

**Figure 5:**
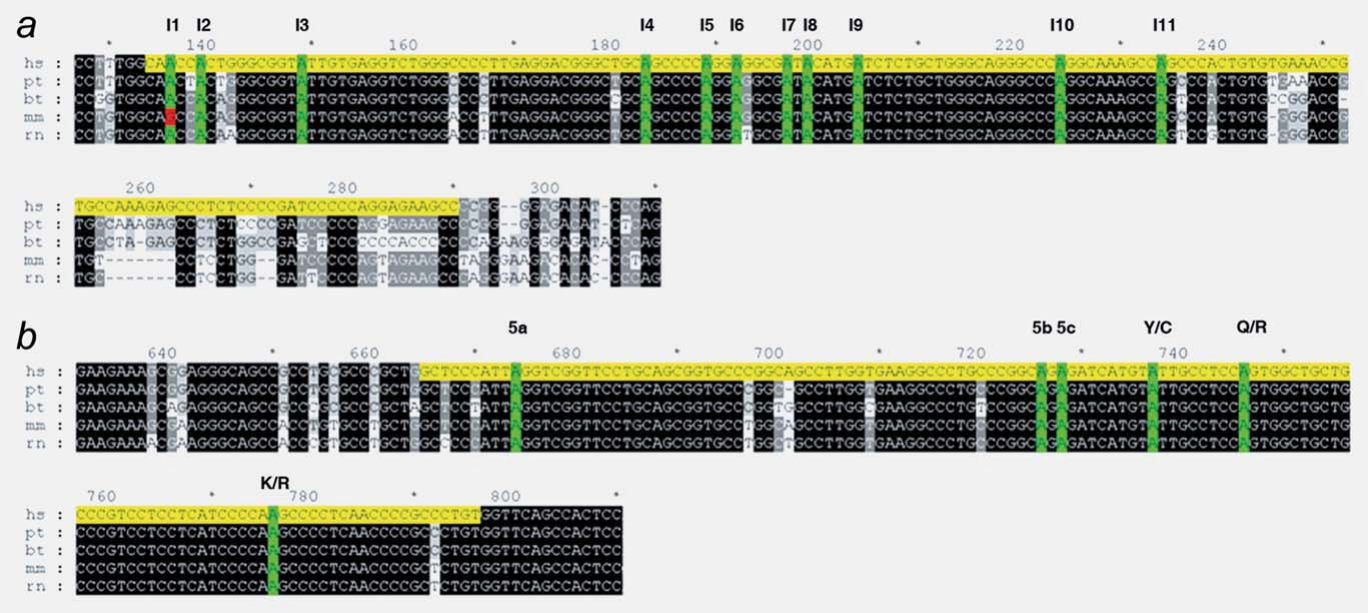
Alignment of *BLCAP* pre-mRNA in mammals. Alignment of *BLCAP* pre-mRNA among mammals identified by initials that correspond to hs, *Homo sapiens*; pt, *Pan troglodytes*; bt, *Bos taurus*; mm, *Mus musculus*; rn, *Rattus norvegicus*. (*a*) Upper alignment corresponds to the last ∼200 nucleotides of the *BLCAP* intron. (*b*) The lower alignment corresponds to the start of exon 2 in *BLCAP*. Nucleotide positions found edited in human are highlighted in green. The first ATG of the coding sequence corresponds to nucleotides 734–736 in (*b*). Sequences predicted to base pair to form an RNA duplex are highlighted in yellow. Identical nucleotides are indicated in black.

The editing sites in the human *BLCAP* transcript are clustered in 2 specific regions in the pre-mRNA. The first region includes the last 150 nucleotides of the intron and the second region includes the beginning of exon 2 (Fig. 1*a*). This observation raised the question if these 2 portions of the *BLCAP* pre-mRNA could form a duplex that would be recognized by ADARs. It has been well established that many A-to-I editing sites occur in clusters that are conserved. The editing complementary sequences (ECSs) form an imperfect duplex with the edited region^35^ and are often found within an intron. As intronic ECS is often conserved among species, we performed multiple alignments of mammalian *BLCAP* pre-mRNA and indeed we found a high level of conservation in the last 150 nucleotides of the intron and the beginning of exon 2 (Fig. 5).

Then, we analyzed the human *BLCAP* pre-mRNA by RNA folding algorithm. A stable secondary structure was predicted to be formed between the intron and the coding region, highlighted in yellow in Figure 5, which includes all the editing sites found in this study.

**Figure 6:**
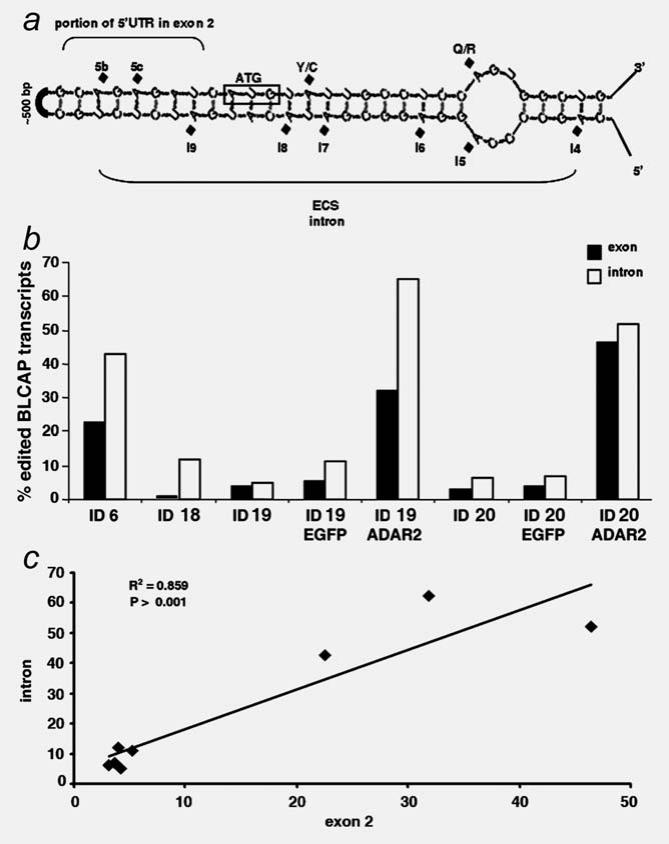
Comparison of editing within the intron and exon of *BLCAP*. (*a*) A portion of the predicted dsRNA structure formed between the intron and exon 2 is shown. The hypothetical ECS is also indicated. (*b*) The editing events present in *BLCAP* mRNA are expressed as a percentage (*y*-axis) and tissues, and cell lines are indicated with their ID identification number in all the samples studied (*x*-axis). The number of transcripts edited in the intron (in white bars) and in the exon (black bars) are shown. (*c*) Correlation of RNA editing between exon 2 and the intron in *BLCAP*. RNA editing within the *BLCAP* intron is expressed as a percentage (*y*-axis) and plotted against RNA editing within the *BLCAP* exon 2 (*x*-axis). Analysis was performed with linear regression; *P* values and *R*^2^ coefficients are shown.

In Figure 6*a* a portion of the predicted dsRNA structure, which includes the editing sites I4–I9, 5b, 5c, Y/C and Q/R, is shown. It is likely that this duplex is required for the editing events found within the intron and the exon of *BLCAP*. If this prediction was true, then the exonic and intronic editing events would be linked and occur simultaneously. We therefore analyzed the editing events in both the exonic and intronic regions of *BLCAP* in normal brain, brain tumor (astrocytoma grade IV) and astrocytoma cell lines untransfected and transfected with the editing enzyme ADAR2 as ADAR2 edited most of the exonic sites studied at a higher level (Tables 1 and 2).

Tissues and cell lines used in this study are identified by progressive identification numbers ID (see Material and methods section). Normal white matter (ID 6) displayed a high level of editing of transcripts edited in both the exon (20%) and the intron (40%) (Fig. 6*b*). However, the corresponding astrocytoma tumor (ID 18) and astrocytoma cell lines U118 and A172 (from now on referred to as ID 19 and 20, respectively) showed a decrease in editing of both exonic and intronic sites with ∼0–5% of transcripts being edited within the exon and 2–10% being edited within the intron (Fig. 6*b*). When we increased the editing activity in these cells by over-expressing ADAR2, we observed an increase in editing in the exon but also in the intron (ID19-ADAR2 and ID20-ADAR2 in Fig. 6*b*).

The relationship between the editing events in exon 2 and the intron of the *BLCAP* transcript in these samples was evaluated with regression analysis. RNA editing in the intron displayed a high correlation with the editing in the exon 2 (*R*^2^ = 0.85) (Fig. 6*c*), suggesting that the editing events in the intron and exon 2 occurred in concert.

### Human *BLCAP* transcript displays diverse editing levels in nervous and non-nervous tissues

As *BLCAP* is expressed in different human tissues, we wanted to determine if the new editing sites found in bladder tissue were present in other tissues and to what extent they were edited. We focused on the editing sites that are present within the mRNA of *BLCAP*. We analyzed the 3 editing events in the 5′UTR (5a, 5b and 5c) and the 3 editing events in the coding sequence (Y/C, Q/R and K/R) in various human tissues and cell lines that are identified by progressive identification numbers (ID). *BLCAP* mRNA was found to be edited in a wide range of human tissues, such as heart, bladder, lymphocytes, fibroblast, epithelial cells and brain (Table 3). However, equivalent editing positions in different tissues were found to be edited to differing extents with the heart and fibroblast mRNAs being edited to a low level at all 6 editing sites and bladder with the highest level of editing (Table 3). Heart and fibroblast displayed the lowest level of RNA editing with 5.1% at the Y/C site, 3.8% at the Q/R site and 1.3% at the K/R site in heart (ID 1) and 7.7% editing at the Y/C and Q/R sites and 0% at the K/R site in fibroblasts (ID 5) (Table 3). Bladder tissue (ID 2) had the highest editing activity with 27.6% at the Y/C site, 15.8% at the Q/R site and 5.3% at the K/R site, respectively (Table 3).

**Table 3:** Editing of *BLCAP* mRNA, within EXON 2 in various human tissues

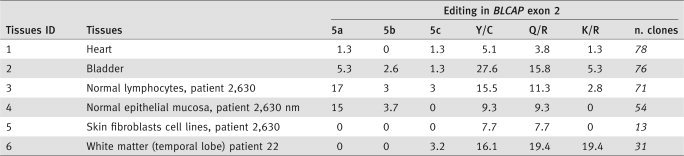

Editing levels are expressed as percentages. In italics is the number of *BLCAP* clones that were sequenced.

It has been shown previously that ADAR1 plays a major role in editing the Y/C site in mouse brain.^42^ In this study, we observed that the Y/C site is edited in human cell lines by both ADAR1 and ADAR2. Moreover we found that the K/R site seems to be preferentially edited by ADAR2 in an overexpressing cell system in HEK 293T and astrocytoma cell lines.

Therefore, we can conclude that ADAR2 appears to be more active in editing this transcript in the brain (K/R site = 19.4%) than in other tissues (Table 3); however, ADAR1 alone or in concert with ADAR2 seems to be more active in editing this transcript in bladder (Y/C site = 27.6%), lymphocytes (Y/C site = 15.5%) and brain (Y/C site = 16.1%) compared with other tissues (Table 3). Real-time experiments on human normal tissues extracted from heart, brain, lymphocytes and bladder (see Table 3) were performed to analyze the ADAR mRNA expression levels. Indeed, we observed that tissues with a high-editing level such as lymphocytes and bladder tissues (Table 3) also displayed a high-expression level of ADAR1 and/or ADAR2 (Fig. 2S). The brain tissue with a high level of edited *BLCAP* transcript showed a low level of ADAR1 mRNA, but this is compensated by a high level of ADAR2 mRNA expression (Fig. 2S). Finally heart tissue with the lowest percentage of *BLCAP* editing level among the tissues tested (Table 3) also showed the lowest level of mRNA expression of both ADAR1 and ADAR2 (Fig. 2S).

### A general decrease in editing activity of the human *BLCAP* transcript in cancerous tissues

As A-to-I editing was found to be downregulated in various tumor tissues compared with the normal control tissues^17^,^18^,^20^ and considering that we observed a decrease in editing level in astrocytoma grade IV and astrocytoma cell lines compared with normal white matter (Fig. 6*b*), we wondered if this would also be observed in tumors from other tissues. We analyzed the editing frequencies at the new editing sites in the *BLCAP* transcript in normal and cancerous tissues and cells lines (Table 4). Despite the small number of patients analyzed in this study, we found a general decrease in editing in the *BLCAP* mRNA in cancerous cell lines (Table 4, samples in dark gray) and cancerous tissues (Table 4, samples in light gray) when compared with normal tissues (Table 4, samples in white). In particular, tissues with a higher level of editing activity such as bladder and brain had a greater decrease in editing at specific sites. In normal bladder tissue (ID 2), editing at the Q/R site was 15.8%, whereas in transitional cell carcinoma (TCC) and bladder cancer cell lines editing at this site ranged from 3.6 to 8.2% (ID 7–9). Analysis of the K/R site revealed editing levels of 5.3% in normal bladder but in bladder cell lines editing at this site ranged from 0 to 1.5% (ID 10–12) and from 3.4 to 8.2% in invasive TCC (ID 7–9) (Table 4).

**Table 4:** Editing level of *BLCAP* mRNA within EXON 2 in cancerous tissues and cell lines and noncancerous tissues

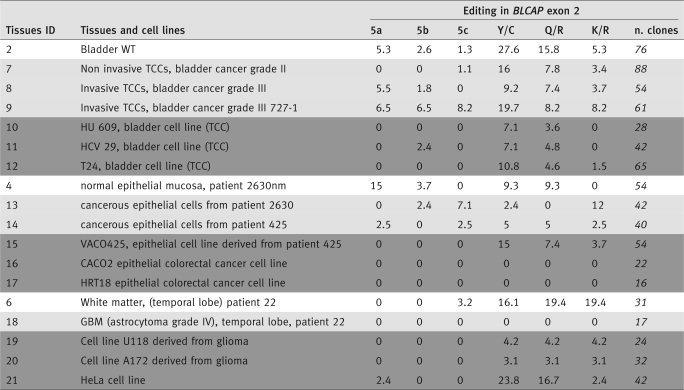

Indicated in white are control human tissues, in gray tumor tissues and in dark gray cancerous cell lines. Editing levels are expressed as percentages. In italics is the number of sequenced *BLCAP* clones.

In normal tissue, editing activity in the white matter (ID 6) was 19.4% at the Q/R site, and this was reduced to 0–4% in cancer tissue and cell lines (ID 18–20). Similarly, the K/R site in white matter was edited to 19.4%, and it decreased to 0–4.2% in tumors and cancer cell lines (ID 18–20).

The editing sites within the 5′UTR were found to be edited to a very low level even in normal tissues (Table 4, samples in white), whereas, in most cancers and cell lines, these editing sites are basically not edited (Table 4, samples in light and dark gray).

Nevertheless, there were some exceptions such as the epithelial colorectal cancer cell line (ID 15) from patient 425 that had 15% editing at the Y/C site, which is higher than the normal control (ID 4) (Table 4).

Furthermore, we analyzed if a correlation exists between grade of malignancy of the tumors and percentage of editing at Y/C, Q/R and K/R sites in 7 pediatric astrocytomas at increasing grade of malignancy from astrocytoma grade I to astrocytoma grade IV also known as glioblastoma multiforme all dissected from the Temporal/Frontal lobe. Despite our small cohort of patients, we found a good correlation between a decrease in editing level at the Q/R and K/R editing sites of *BLCAP* and the increase of the histological malignancy of the tumors (Fig. 3S), however, poor correlation was observed for the Y/C editing site (Fig. 3S).

## Discussion

BLCAP is a highly conserved protein present in all species from *C. elegans* to humans. Despite its high protein conservation (Fig. 2), the function of this protein is still unknown. Multiple alignments of *BLCAP* pre-mRNA from different species show that conservation extends into the RNA level including noncoding regions such as the 5′UTR and the unique intron (Fig. 5). As UTRs and introns in eukaryotes are generally under less selective pressure than the coding regions,^43^ the conservation of these regions in *BLCAP* pre-mRNA may reflect the presence of specific consensus sequences that are important for posttranscriptional regulation of the transcript such as RNA editing. In fact, a duplex RNA structure comprising of the conserved intronic region and the exon 2 was predicted (Fig. 6*a*). Double-stranded RNAs are indeed substrates for ADAR enzymes and, as expected, the human *BLCAP* pre-mRNA undergoes multiple editing events in the predicted dsRNA structure.

A total of 17 editing sites were found in the human *BLCAP* pre-mRNA (Fig. 1). Eleven editing sites were identified within the intron (I 1–11), 3 within the 5′UTR (5a–c) and 3 in the coding sequence (Y/C, Q/R and K/R) (Fig. 1). The 3 editing events within the coding region of *BLCAP* can change the amino acid composition of the protein, thereby generating up to 8 different BLCAP protein isoforms. As the coding region of this gene is intronless (the only intron lies within the 5′UTR), RNA editing is an important way to generate protein isoforms. Importantly, the 3 editing events occurred within the coding region of the *BLCAP* mRNA lying within the most highly conserved region of the protein within the N-terminus before the hypothetical first TM1. These editing events (Q/R and K/R) introduced a positively charged amino acid. The new Q/R editing event, found in this study, generates an important amino acid change as it substitutes an uncharged residue (Q) with a positively charged and bigger residue (R). Interestingly, the other new K/R editing site, that also introduced an arginine, lies within the hypothetical proline-rich domain at the N-terminus of the protein (Fig. 2). The proline-rich domain is commonly found in proteins involved in cytoskeleton rearrangement, signaling cascades and initiation of transcription.^44^,^45^ As the biological role of this protein is still unknown, we can only speculate on the possible consequence of the editing events.

As *BLCAP* is edited at multiple sites within its coding regions and it is abundantly expressed in brain, it was likely that ADAR2 could play a major role in editing this transcript. However, it seems that both ADAR enzymes edit *in vivo* the Y/C site previously identified^42^ with a major contribution due to ADAR1.^42^,^46^ We confirmed, using a different system, that both ADAR1 and ADAR2 can edit the Y/C site (Table 1). We analyzed editing of the endogenous *BLCAP* transcript in a cell line in which we overexpressed either ADAR2 or ADAR1. In this assay, we found that in HEK 293T both editing enzymes can edit *BLCAP* transcripts to a varying extent (Table 1). Interestingly, the K/R site appears to be preferentially edited by ADAR2 (Table 1).

As *BLCAP* is expressed in different human tissues, we investigated differences in editing occuring in different tissues. Interestingly, equivalent editing positions in different tissues were found to be edited to differing levels with the heart and fibroblast mRNAs being edited at a very low level, however, bladder, brain and lymphocytes showed a higher editing activity (Table 3), and the consequence of this would be that different ratios of BLCAP protein isoforms is present in different tissues.

As *BLCAP* transcript is ubiquitously expressed and edited to varying extents in different tissues, it could be useful as a comparative editable transcript for further studies on the fine tuning and regulation of ADARs editing activity *in vivo*.

Furthermore, we also tested the editing level of *BLCAP* in a cohort of patients, and we found a general decrease in editing activity in tumors *versus* tissues of non-tumoral origin. In particular we analyzed astrocytomas, bladder carcinoma and colorectal carcinoma (Table 4).

This observation is of interest as there is growing evidence showing an overall downregulation of A-to-I RNA editing in tumor tissues when compared with control tissues not only in brain^18^,^20^ but in different tumor tissues such as placenta, testis, kidney, lung, prostate and muscle.^17^ The reason why ADARs have different activities in tumor tissues *versus* controls is not yet fully understood. Nevertheless, it has been reported that altered ADARs mRNA levels is present in brain tumors compared to normal tissues.^17^,^18^ However, it is unlikely that the expression level of ADARs proteins is the only parameter that can explain the modulation in editing in normal *versus* pathological conditions. Indeed, it has been recently shown that ADARs activity can be influenced by the presence of a regulatory network of proteins and/or RNAs that could modulate their function.^5^,^18^,^47^–^50^ *BLCAP* was first identified in a screen for genes contributing to the invasive phenotype of renal, cervical and bladder cancer (TCC) as its transcript was found to be downregulated in these tumors.^28^,^30^,^31^ Furthermore, herein, we found that BLCAP edited isoforms are reduced in different type of cancers. In addition, despite our small cohort of tumors samples, we observed that a correlation exists between a decrease in ADAR-mediated RNA editing on *BLCAP* transcript and the histological grade of malignancy in pediatric astrocytomas (Fig. 3S).

Therefore, in cancers, *BLCAP* transcript is not only downregulated but is also less edited. This study opens up an additional question to be addressed by future work as to whether the editing events identified herein can contribute to the appearance or progression of different human cancer.

A recent work reported no significant changes in editing levels between normal tissues and urinary bladder neoplasm.^51^ Differences existing between this observation and this study could be due to different patients enrolled and/or different methods used to identify editing events.

In conclusion, we identified new editing sites within the human *BLCAP* transcript that required both ADAR1 and ADAR2. The editing sites in different human tissues are differently edited. Furthermore, the finding that *BLCAP* editing is downregulated in various cancerous tissues could be valuable for future improved diagnosis and therapies.
