# Supplementary figures and images for: Human BLCAP transcript: new editing events in normal and cancerous tissues
Source: Int J Cancer. 2009 Nov 11;127(1):127–37. doi: 10.1002/ijc.25022 (PMC2958456; doi:10.1002/ijc.25022)

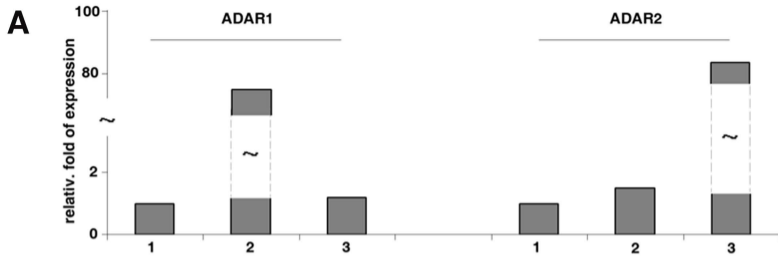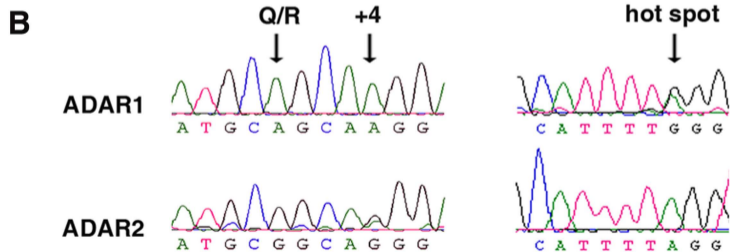

Figure 1S

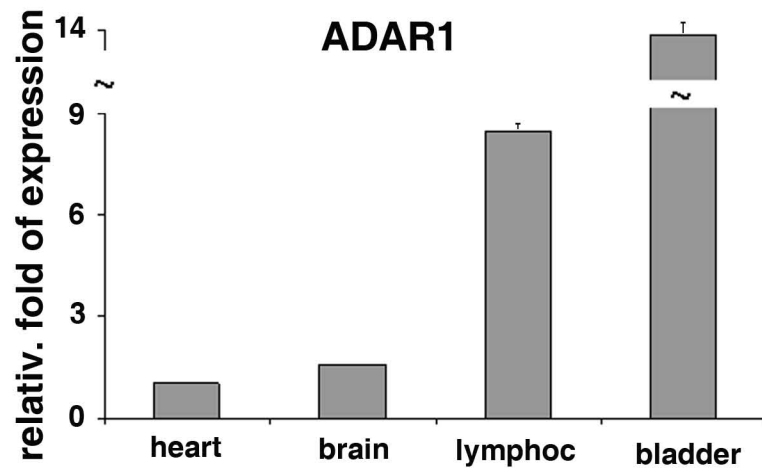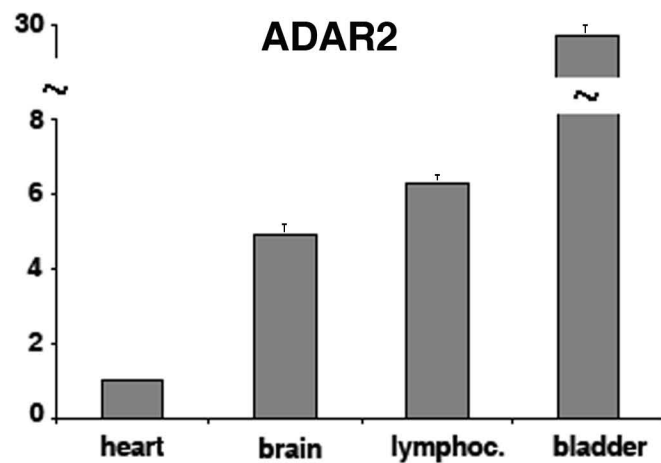

Figure 2S

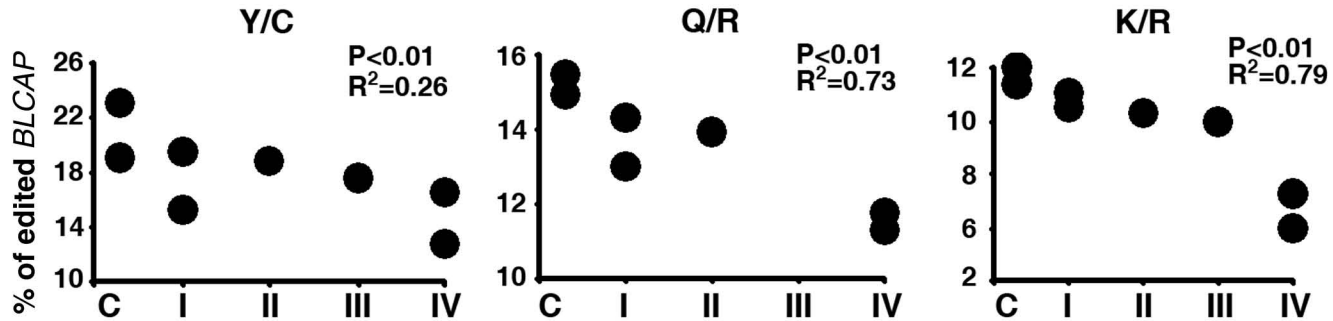

Figure 3S

Supplement: Supplementary file 1 [file ijc0127-0127-SD1.pdf]
